# Initial Experience with High-density Mapping of Ischemic Ventricular Tachycardia Using a Narrow 0.1-mV to 0.25-mV Border-zone Window

**DOI:** 10.19102/icrm.2020.111006

**Published:** 2020-10-15

**Authors:** Felix Yang, Jordan Roy, Abhinav Saxena, Guy Kulbak, Yisachar Greenberg

**Affiliations:** ^1^Maimonides Medical Center, Brooklyn, NY, USA

**Keywords:** Ablation, high-density mapping, ventricular tachycardia

## Abstract

This study sought to determine (1) whether the use of a narrow border-zone voltage of 0.1 to 0.25 mV predicts the ventricular tachycardia (VT) exit site better than when using the conventional 0.5 to 1.5 mV window and (2) the feasibility of utilizing the Rhythmia mapping system (Boston Scientific, Natick, MA, USA) to map hemodynamically unstable VT without hemodynamic support. The Ablation of ischemic VT is challenging especially in the setting of hemodynamic instability, yet efficient and accurate mapping of VT and VT substrate is critical for procedural success. In this study, a total of 24 patients with ischemic cardiomyopathy and recurrent monomorphic VT underwent mapping and ablation using the Rhythmia system. Contact-force sensing ablation catheters were use in two cases. In patients with mappable VTs, the distance between the exit site and border zone was calculated for border zone-voltage windows of 0.5 to 1.5 mV and 0.1 to 0.25 mV. The percentage of LV scar for each patient was visually estimated into quartiles of scar burden in both windows. Twenty patients were inducible into VT, while 15 patients had mappable VTs for a total of 16 VTs (11 stable VTs and five unstable VTs). There were no adverse complications in patients who underwent mapping in unstable VT. The mean distance from the VT exit site to the border zone was 13.3 mm in the conventional window and 3.4 mm in the narrow window (95% confidence interval: 4.0–15.8; p = 0.003). Separately, 94% (15/16) of the VTs were mapped to the narrow border-zone voltage versus 31% (5/16) using the conventional border zone (p = 0.0006). The use of a narrow 0.1- to 0.25-mV border-zone window highlights relevant scar and constitutes a border zone where VT exit sites are frequently located. We also found that exit sites of hemodynamically unstable VTs can be identified without an increase in procedural complications using the Orion catheter (Boston Scientific, Natick, MA, USA).

## Introduction

The ablation of ventricular tachycardia (VT) may be conducted using a number of different strategies. In addition to VT mapping, the delineation of dense scar and the border zone are key aspects of successful VT ablation. The Rhythmia mapping system (Boston Scientific, Natick, MA, USA) facilitates rapid high-density mapping of VT and VT substrate. Since VT channels are frequently located at very low voltages that have traditionally been defined as scar^1,2^ and since most VT channels have been found to be located in scar between voltages of 0.2 mV to 0.3 mV,^3^ we sought to determine whether the use of a narrow border zone voltage of 0.1 mV to 0.25 mV predicts the VT exit site better than the conventional 0.5-mV to 1.5-mV window. Additionally, since VT is often hemodynamically unstable, we investigated whether mapping during short periods of hemodynamic instability using the Orion catheter (Boston Scientific, Natick, MA, USA) would allow the operator to determine VT exit sites.

## Methods

### Study population

The study population consisted of patients with ischemic cardiomyopathy and recurrent monomorphic VT who underwent mapping and ablation with the Rhythmia system. This study was approved by the institutional review board of Maimonides Medical Center, which waived the need for informed consent.

### Ablation procedure

Patients underwent deep sedation and/or general anesthesia per the operator’s discretion. Mapping was performed using the Orion mapping catheter (Boston Scientific, Natick, MA, USA). Patients underwent transseptal puncture to gain access to the left ventricle. Mapping was performed with the patient in a sinus- or ventricular-paced rhythm to obtain a left ventricular geometry and voltage map. Induction was completed using conventional ventricular programmed electrical stimulation and/or ventricular ultrarapid trains. Conventional programmed electrical stimulation was performed at multiple drive trains down to ventricular refractoriness to triple extrastimuli at two sites. Ultrarapid trains were performed at multiple drive trains to double extrastimuli at two sites.^4^ VT was mapped with the patient in tachycardia when hemodynamically stable. Unstable VT, defined as VT where the systolic blood pressure was less than 80 mmHg, was mapped in up to one-minute intervals with the Orion catheter and then pace-terminated or cardioverted to baseline rhythm. Pacemapping was performed as needed to aid in the localization of a VT exit site. Ablation was performed at up to 40 W with an irrigated ablation catheter for up to one-minute intervals. Scar homogenization was performed in the regions of the scar and border zone as defined by the 0.1-mV to 0.25-mV voltage window. Reinduction of VT was completed until no further VT was inducible. The procedure was terminated without the achievement of noninducibility if there were concerns of sustained hemodynamic instability or prohibitive procedural duration.

### Clinical follow-up

Patients were followed up by implantable cardioverter- defibrillator (ICD) interrogation every three months postprocedure. Remote follow-ups were reviewed for VT recurrence. Recurrent VT/ventricular fibrillation (VF) was defined as documented VT/VF lasting longer than 30 seconds or requiring any appropriate ICD therapy including antitachycardia pacing. Antiarrhythmic use after ablation was decided at the discretion of the treating physician.

### Statistical analysis

In patients who had mappable VTs, the distance between the exit site and the border zone was calculated for border-zone voltage windows of 0.5 mV to 1.5 mV and 0.1 mV to 0.25 mV. The percentage of LV scar burden in each person was visually estimated into quartiles of scar burden using the 0.5-mV to 1.5-mV window and compared with that of the 0.1-mV to 0.25-mV window. Continuous variables are expressed as mean ± standard deviation and were compared using Student’s t-tests. Categorical comparisons were performed with Fisher’s test. Statistical analysis was conducted using the Stata version 14.2 software program (StataCorp, College Station, TX, USA). The significance level was set at 0.05.

## Results

Baseline characteristics of the study participants are shown in **Table 1**. Of 24 patients who underwent mapping and ablation of ischemic VT using the Rhythmia system, 20 patients were inducible into VT. Meanwhile, 15 patients had VTs that were mappable with the Orion catheter, for a total of 16 VTs. Eleven mapped VTs were hemodynamically stable and mapped during the arrhythmia. Mapping during unstable VT determined VT exit sites in five patients for five VTs. The mean rate for stable VT was 132 bpm ± 13 bpm, while the mean rate for unstable VT was much faster at 185 bpm ± 11 bpm (p < 0.0001). The mean mapping times in stable and unstable VT were 508 seconds and 294 seconds, respectively (p = 0.29). There were no adverse complications in patients who underwent mapping in unstable VT.

Ablation was performed using a Boston Scientific (Natick, MA, USA) non–force-sensing irrigated-tip ablation catheter in 22 of 24 patients. Ablation using the Tacticath SE ablation catheter (Abbott Laboratories, Chicago, IL, USA) was performed in two of 24 patients. One patient died of pneumonia sepsis in the index hospitalization period. Of the remaining study population, 17 (74%) of 23 patients were free from VT/VF during follow-up. Patients who had VTs mapped experienced a 14% (2/14) rate of recurrence versus the 44% (4/9) rate of recurrence see in nonmapped patients at a mean follow-up of 881 days. One patient who initially underwent ablation of a mapped VT using a non–contact-force ablation catheter underwent successful reablation with a contact-force ablation catheter deployed in the same region due to VT recurrence; this procedure successfully eliminated any VT during follow-up. Both patients who were ablated using contact-force ablation catheters during the index procedure did not experience any VT recurrences during follow-up.

While 17 patients were on antiarrhythmic therapy preablation, only five patients were on antiarrhythmic therapy at last follow-up. None of the patients with mapped unstable VT experienced recurrence. Further, none of the 24 included patients experienced complications such as stroke, hematoma, major bleeding, pulmonary embolism, deep venous thrombosis, cardiac perforation, or cardiac tamponade as a result of their procedure.

**Figure 1:**
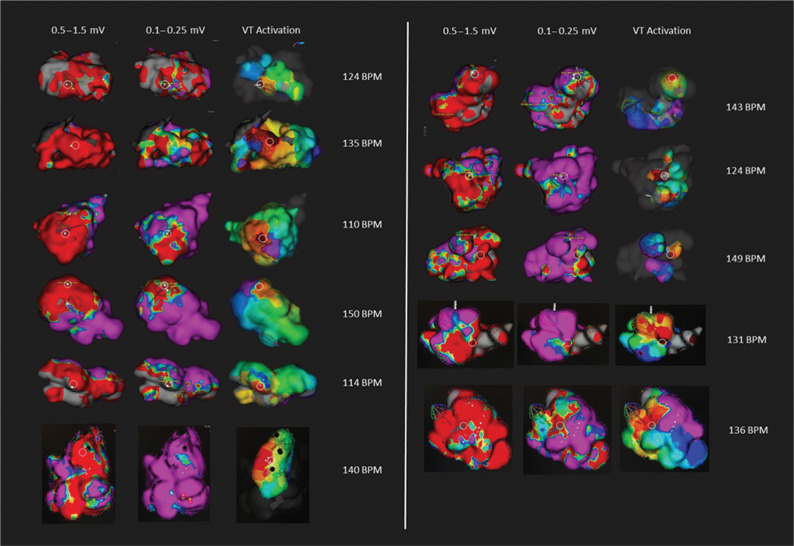
Stable VT maps: comparison of voltage maps using conventional versus narrow voltage windows and VT activation. Dense scar is depicted in red; the border zone is depicted in yellow, green, and blue; and normal myocardium is depicted in purple. VT activation is depicted in the right column with the exit sites in red. In the first two columns, the white circles indicate the VT exit sites in each patient on the voltage maps. For the majority of these patients, the VT exit sites are deep within dense scar when the scar is defined as less than 0.5 mV. However, using the narrow voltage window of 0.1 mV to 0.25 mV, most of the patients’ VT exit sites are in fact located in the border zone voltage. The use of the narrow voltage window results in a smaller region of the left ventricle being defined as dense scar. This may translate to more efficient targeting of critical isthmus and exit sites during ablation.

**Figure 2:**
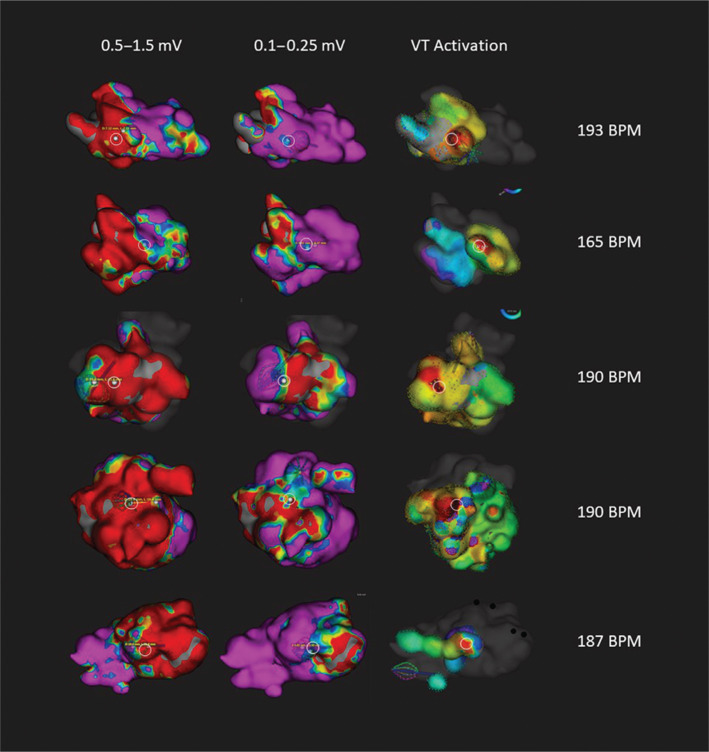
Unstable VT maps: comparison of voltage maps using conventional versus narrow voltage windows and VT activation. Despite faster VT cycle lengths and greater hemodynamic instability, VT exit sites were able to be determined in five patients. The exit sites (white circles) correspond to narrow window border-zone voltages in these patients. Scar maps with conventional (0.5–1.5 mV) and narrow (0.1–0.25 mV) voltage windows are depicted in the first two columns. VT activation maps are depicted on the right, with the VT exit sites in red.

The mean distance from VT exit (white circles in **Figures 1 and 2**) to the border zone was 13.3 mm in the conventional window map and 3.4 mm in the narrow window map (95 confidence interval: 4.0–15.8; p = 0.003). Of the 16 mapped VTs, 15 had exit sites that corresponded to the narrow border-zone window voltages. In contrast, only five of the 16 mapped VTs had exit sites in the conventional border-zone window. The remaining 11 of 16 patients showed VT exit sites emanating from dense scar according to the conventional border-zone window. Using the conventional window, four patients showed less than 25% scar, 13 showed 25% to 50% scar, one showed 50% to 75% scar, and six showed greater than 75% scar. Using the narrow window, 18 patients experienced a reduction in the scar quartile. One patient’s scar zone was initially defined as greater than 75% of the LV with the conventional window but was reduced to less than 25% of the LV with the narrow window.

## Discussion

The ablation of ischemic VT is made challenging by a variety of factors. The VT substrate is frequently approachable from an endocardial route; however, VT circuits may be midmyocardial or more epicardial in origin and not readily treatable from a simple endocardial route. Patients may have a large region or multiple patches of scar and multiple VT exit circuits. VT may or may not be inducible during the time of ablation and, if induced, may not be mappable as a result of hemodynamic instability. Finally, ischemic VT patients are generally those that have reduced ejection fractions and multiple comorbidities, putting them at increased risk for procedural and anesthesia complications.

There are a number of methods by which to approach ischemic VT substrate: scar homogenization,^5^ scar dechanneling,^6^ targeting late potentials and local abnormal ventricular activities,^7^ and core isolation.^8^ Each method attempts to eliminate critical regions of ventricular scar and ultimately achieve noninducibility of VT. Mapping during VT facilitates the direct assessment of a VT exit site and, with the advent of high-density mapping, conduction through a critical isthmus. Unfortunately, many VTs are hemodynamically unstable and mapping in cases of continuous VT may require hemodynamic support or be impossible.

The advent of the Orion high-density basket catheter has supported the rapid assessment of ventricular scar as well as ventricular activation in VT.^9,10^ The speed and accuracy of point acquisition inherent with this device enable an operator to map in patients with unstable VT over short periods of time. In our series of patients, the majority of patients (15/24) had VTs that were able to be mapped. Eleven of the mapped VTs were hemodynamically stable during mapping **(Figure 1)**, whereas five were unstable **(Figure 2)**. The mean rate for unstable VT was significantly faster than that of stable VT (185 ± 11 versus 132 ± 13 bpm; p < 0.0001). In our practice, we found that patients tolerated VT hemodynamically better with deep sedation rather than with general anesthesia and adopted this approach in the majority (17/24) of cases.

A number of lessons were learned during our experience with high-density left ventricular voltage mapping. Initial voltage maps are created during right-ventricular pacing to allow for better appreciation of late regions of activation. This prevents the collision of wavefronts from normal Purkinje activation in normal sinus rhythm or biventricular activation in cardiac resynchronization therapy pacing. A propagation map during normal sinus rhythm extending to late diastole allows for late potentials to be identified. Limiting the propagation map to a post-QRS window is also useful in situations where there are multiple near-field and far-field signals. Since late activation is often seen as low-voltage fractionated signals, the default 0.03-mV confidence mask may need to be lowered to 0.01 mV. All of our patients were mapped using a transseptal approach and a large-curl Agilis sheath (Abbott Laboratories, Chicago, IL, USA). We found that this allowed us to reach most areas of the left ventricle efficiently and in a stable manner. Scar maps were examined in the conventional 0.5-mV to 1.5-mV border-zone range and then in the 0.1-mV to 0.25-mV border-zone range. Invariably, the narrower range yielded a smaller scar from which we initially targeted VT mapping and ablation.

While mapping during VT, the Orion basket catheter was initially placed in proximity to regions of scar and particularly late potentials. This resulted in a higher chance that it would map salient components of the VT cycle length early on. The Orion catheter was then moved toward a more proximal (red) zone of the activation map to quickly obtain the exit site.

**Figure 3:**
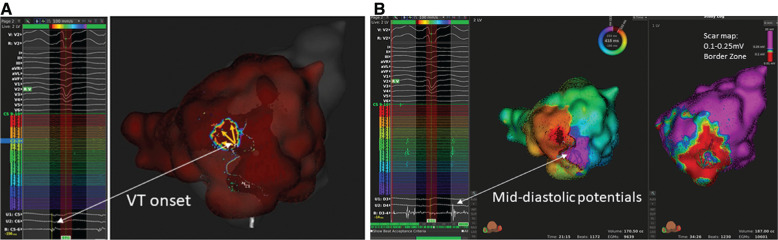
Sunburst pattern of VT exit and diastolic potentials. **A:** VT exit in a patient with a typical sunburst appearance. The electrogram at the region of onset is pre-QRS. **B:** Mid-diastolic potentials, which are located in the scar zone just proximal to the VT exit site.

The propagation maps of the VT frequently demonstrated a “sunburst” pattern of VT exiting the critical isthmus **(Figure 3)**. Ablation was performed at the region of the exit site and the region of scar proximal to the exit site. Termination during VT was usually noted at these proximal regions. We also observed that VT exit sites tended to exist at or near the 0.25-mV range as depicted by a green-blue color on the voltage map if one uses a 0.1-mV to 0.25-mV border-zone range. Additionally, late potentials frequently were identified in these same regions. Ablation at these areas and the dense scar located proximally appears to be of high yield.

Notwithstanding one patient who underwent general anesthesia and expired due to pneumonia sepsis during the index hospitalization period, there was a 74% rate of freedom from VT at a mean of 881 days of follow-up. Among the cases of recurrences, two patients experienced VT storm, for which ICD shock therapy was required. Encouragingly, there was an 86% rate of freedom from VT when the patient’s VT was able to be mapped. Most patients were also off antiarrhythmic therapy during follow-up. What then limited the mapping of the patients’ VT? Some patients (4/24) were not able to be induced into VT at the time of mapping possibly due to the undesirability of discontinuing antiarrhythmic therapy given the history of prior VT therapies. Five additional patients were inducible into VT but were not mapped due to hemodynamic or other operative concerns precluding mapping and ablation in VT.

The Orion mapping catheter was a critical tool in VT substrate and tachycardia mapping, especially given that 21% (5/24) of patients were mapped during unstable VT. The identification of the VT exit and circuit allowed for a targeted ablation while demonstrating the utility of the 0.1-mV to 0.25-mV border zone in predicting VT exits and regions of late potentials. Of all mapped VTs, 94% (15/16) were mapped to the narrow border-zone window voltages. In contrast, if one were to consider the conventional border-zone voltages, only 31% (5/16) of the exit sites were mappable to conventional border-zone regions, while the remaining 69% (11/16) appeared to be buried in deep scar. The use of the narrow border window may allow for a more efficient targeting of critical scar zones when VT cannot be induced or mapped.

Since there is a high prevalence of abnormal substrate in the endocardial regions of ischemic VT substrate, we surmise that this narrow voltage window helps to delineate endocardial dense scar from regions of low-amplitude fractionated signals that are frequently found near VT exit sites. This window allowed us to focus our ablation in regions at the exit site and just proximal to them in the scar to achieve a high rate of success. The discrepancy in what is defined as scar using the conventional and narrow voltage windows may perhaps reflect scar that is deeper, midmyocardial in nature, or of ventricular tissue, which is not as prone to conduction delays that are supportive of VT reentry. In the patients who were not able to undergo VT mapping, ablation was performed empirically using pacematching and scar homogenization with the narrow window; however, the success rate was not as high. This could reflect deeper substrate, unaddressed substrate beyond the narrow window, or an inadequate delivery of energy in critical regions of the scar due to a lack of contact force.

## Limitations

Ablation success may be limited partially due to the current lack of force-sensing catheters to pair with the Rhythmia mapping system. Only two of the patients in this series were ablated with force-sensing catheters using the Tacticath SE ablation catheter in a cross-platform arrangement. Presumably, better contact with the endocardium would translate to improved lesion depths and better outcomes. This would particularly be of use in patients with critical circuits located deeper into the myocardium. The applicability of the narrow voltage window may not be possible in patients with nonischemic cardiomyopathy, as the patients examined in this study had ischemic heart disease.

## Conclusion

In our experience, the exit sites of mapped VTs were more frequently found to exist at the border zone of this narrow 0.1-mV to 0.25-mV window rather than the conventional 0.5-mV to 1.5-mV scar window. Use of the Orion catheter also enabled mapping during hemodynamically unstable VT without an increase in procedural complications. Although we recorded a very low postprocedure recurrence rate when VT exit sites were identified, we anticipate that our success rates would increase with the advent of a navigation-enabled contact-force ablation catheter paired with the Rhythmia system. We found that high-density mapping of VT allowed us to quickly determine VT exit sites and VT substrate, enabling us to focus more on the therapeutic aspect of ablation. Additional clinical and pathologic studies are needed to confirm these findings and further explore the use of high-density mapping in VT.

## Figures and Tables

**Table 1:** Baseline Demographics

Age	71 ± 8 years
Male sex	100%
Mean LVEF	25% ± 9%
NYHA functional class	
I or II	35%
III or IV	65%
Hypertension	85%
Diabetes	55%
ESRD	5%
Prior CABG	80%
Antiarrhythmic therapy	80%
VT storm	85%

CABG: coronary artery bypass graft;

ESRD: end-stage renal disease; LVEF: left ventricular ejection fraction;

NYHA: New York Heart Association;

VT: ventricular tachycardia.
